# Enhancement of the Wear Resistance and Microhardness of Aluminum Alloy by Nd:YaG Laser Treatment

**DOI:** 10.1155/2014/842062

**Published:** 2014-07-17

**Authors:** Haitham T. Hussein, Abdulhadi Kadhim, Ahmed A. Al-Amiery, Abdul Amir H. Kadhum, Abu Bakar Mohamad

**Affiliations:** ^1^Applied Science Department, University of Technology, Baghdad 10066, Iraq; ^2^Laser and Optoelectronic Engineering Department, University of Technology, Baghdad 10066, Iraq; ^3^Department of Chemical and Process Engineering, Faculty of Engineering and Built Environment, Universiti Kebangsaan Malaysia, 43600 Bangi, Selangor, Malaysia

## Abstract

Influence of laser treatment on mechanical properties, wear resistance, and Vickers hardness of aluminum alloy was studied. The specimens were treated by using Nd:YaG laser of energy 780 mj, wavelength 512 nm, and duration time 8 ns. The wear behavior of the specimens was studied for all specimens before and after treatment by Nd:YaG laser and the dry wear experiments were carried out by sing pinon-disc technique. The specimens were machined as a disk with diameter of 25 mm and circular groove in depth of 3 mm. All specimens were conducted by scanning electron microscopy (SEM), energy-dispersive X-ray florescence analysis (EDS), optical microscopy, and Vickers hardness. The results showed that the dry wear rate was decreased after laser hardening and increased Vickers hardness values by ratio of 2.4 : 1. The results showed that the values of wear rate for samples having circular grooves are less than samples without grooves after laser treatment.

## 1. Introduction

The solid surface hardening by laser treatment represents the structural transformations of the material; this can be established by irradiating the surface with a laser pulse. Surface heat treatment with laser beam uses the characteristics of self-quenching that cools rapidly into materials without cooling water unlike general surface heat treatment [1, 2]. If surface of materials is hardened, abrasion resistance and corrosion resistance are increased because a dense and homogeneous structure is formed on the surface. Accurate analysis is needed because the mechanical characteristics of material are changed by scanning laser energy. Surface hardening by laser has two advantages. First, there is no much deformation of material since input energy is not too much. Second, the surface of material is smooth after laser hardening so it does not need postprocess [3, 4]. The surface hardness of tools is an essential element in quality and productivity control of the parts. High strength, toughness, abrasion resistance, and corrosion resistance are needed for high machining speed and high performance parts [5, 6]. Laser processing provides precision of operation, short processing time, and local treatment. Moreover, introducing hard particles such as carbides at the surface during laser controlled melting further improves the microhardness of the surface [7]. Laser hardening is a method in which the high-power laser beam quickly irradiates the specimen surface to increase rapidly the specimen surface temperature that is higher than the phase-transformation point and lower than the melting point. After the laser beam is switched off, the cooling base quickly cools the heated region to quench by itself so that the specimen surface is hardened and its performance is modified and improved. Because there is a smaller heat influence region, its heat distortion is small. We can control the surface temperature and hardening depth by adjusting laser beam output power, laser beam moving velocity, and the diameter of laser beam spot. Laser hardening can improve the specimen surface rigidity, wear ability, toughness, and a lot of mechanical performance. It can realize some special effects that are difficult or not attained with the general heat treatment technique and enhance the specimen lifetime greatly [8]. Surface hardness is inversely proportional to processing speed and laser spot size where it was increased with decreasing either processing speed or laser spot size. However, small laser spot size-high processing speed combination is effective at low laser power range. Large laser spot size-low processing speed combination is effective at high laser power range [9]. In continuation of previous studies [10–18] on corrosion, we have focused on the potential of laser transformation hardening and laser treatment on two types of samples with and without grooves in samples' surface of aluminum alloy.

## 2. Experimental

### 2.1. Sample Preparation

Aluminum alloy samples were prepared and divided into two groups: the first group operated in the form of a cylinder diameter of 25 mm and a thickness of 4 mm and a second group is also operated in the same shape and dimensions but were drilled three circular grooves on the samples surface in depth of 2 mm to reduce the contact area. All samples were cleaned and polished and then washed with HCl and NaOH with four molarities. Finally the samples immersing in distilled water and drying carefully.

### 2.2. Laser Surface Hardening

**Figure 1:**
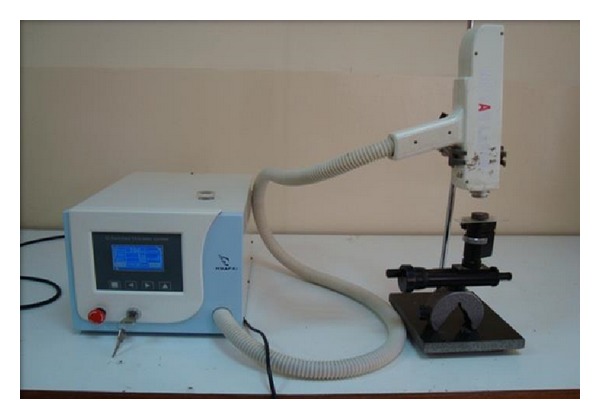
Nd:YaG laser system.

Laser treatment of samples' surface was performed for two types of samples (with grooves and without grooves) by using Nd:YAG pulses laser type Rofin DY044 (as shown in Figure 1) with energy of 700 mj, wavelength of 512 nm, and duration time of 9 ns. The laser beam was guided by a fiber optics to the focusing the laser beam diameter of 4 mm on the surface of sample, being moved over the surface to obtain 4 mm wide tracks. In order to treat wider zones, different tracks were overlapped with an overlapping percentage of 75%. In this case surface treatment by 500 laser pulses for each sample with surface area of 5.09 cm^2^. These samples immersed in distilled water with depth of 3 cm and all samples are dried before starting the experiments of wear.

### 2.3. Composition Analysis

The X-ray fluorescence (XRF) model (TSMJ) made in Germany has been analyzed for aluminum alloy samples to determine the elemental composition of samples.

### 2.4. Wear and Vickers Hardness Experiments

#### 2.4.1. Wear Testing

Pin on disk was used to conduct the dry wear experiments and the wear rate was measured by using the weighing method, and each specimen was weighed before and after wear testing by using sensitive digital balance of accuracy 1 mg. Wear rate values were calculated by using the following equations:

(1) $$\begin{array}{c} \text{wear}\;\text{rate}=\frac{\text{DW}}{\text{SD}}\;\left(\text{gm}/\text{cm}\right),\\ \text{DW}=W_{1}-W_{2}, \end{array}$$

where DW is difference in weight (gm) before and after wear *W*_1_ and *W*_2_ is weight of specimen before and after the test (gm):

(2) $$\begin{array}{c} \text{SD}=2\pi \cdot r\cdot n\cdot t, \end{array}$$

where SD is sliding distance (cm), *r* is radius from the center of the specimen to the center of the disc (cm), *n* is nimber of rotating disc (r.p.m), and *t* is time of sliding (min).

#### 2.4.2. Vickers Hardness Testing

Hardness type Vickers was conducted for all samples before and after laser surface treatment by using (Hensddt Wetzlar), with applied load 500 gm. Vickers hardness values were calculated according to the following equation:

(3) $$\begin{array}{c} \text{ HV }=1.8544\;\frac{F}{d^{2}},\;\left(\text{kg}\;\text{f}/{\text{mm}}^{2}\right), \end{array}$$

where *F* is applied load (kg f) and *d* is the main diagonal of indentation (mm).

## 3. Results and Discussion 

### 3.1. XRF Results

The elemental composition of aluminum alloy samples used in this work was made by using X-ray fluorescence (XRF) analysis technique as shown in Table 1.

**Figure 2:**
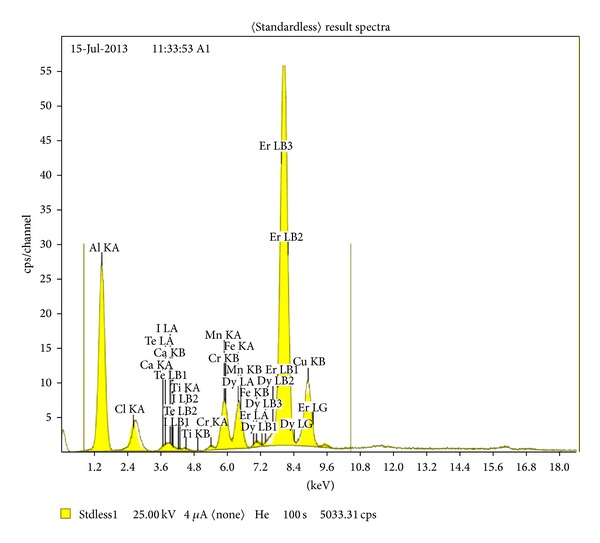
XRF for Al alloy samples.

Figure 2 shows the XRF chart of the elemental composition of aluminum alloy samples. The result reveals different concentrations of lements and the aluminum represents the balance. Concentrations of the very few represents traces or contaminations. This alloy is called local aluminum.

### 3.2. Vickers Hardness

Vickers hardness results of aluminum alloy samples before and after laser surface treatment increased from 98 HV to 235 HV. This enhancement of surface hardness after laser treatment can be represented by the ratio of 235%. Vickers hardness measurements included three readings and then taken average.

### 3.3. Wear Rate Results

**Figure 3:**
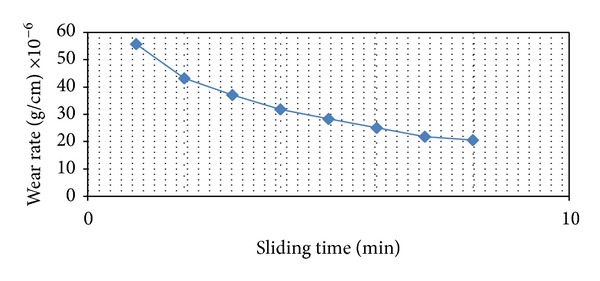
Wear rate (gm/cm) versus sliding time before laser surface treatment under load of 5 N.

**Figure 4:**
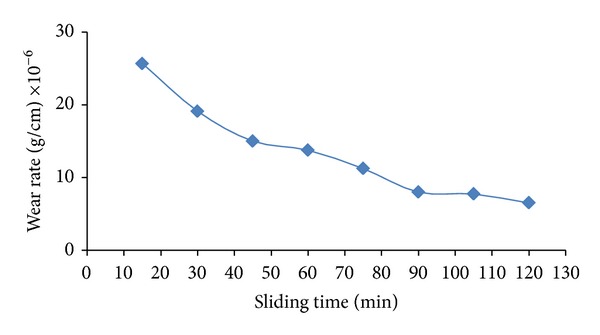
Wear rate versus sliding time after laser surface treatment under load of 5 N and 500 pulses.

Figures 3 and 4 show the relation between the dry wear rate and the sliding time under constant load of (5 N). One can be shown that the wear rate reduced after harding by laser because the hardness values for all samples have been increased after treated by laser; this behavior due to the energy is transferred to the metal, and the material surface originates a phase transformation from solid to vapor state. The plasma is an outcome of the gas phase, which absorbs the energy directly from the laser radiation.

**Figure 5:**
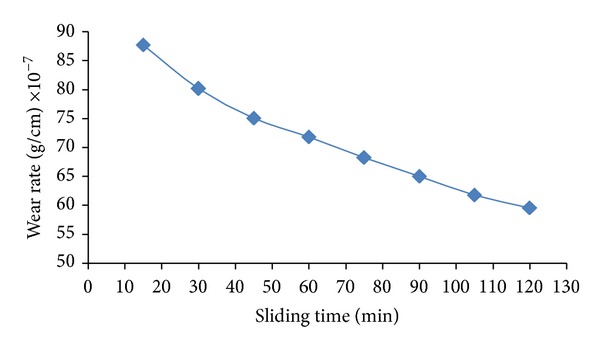
Wear rate versus sliding time after hardening by laser (250 pulses) and groves on surface.

The plasma causes a shock wave by its expansion; this shock increased by distilled water pressure that covers sample during shooting laser pulse. For this reason the hardening was increased and the grain size decreased.  Figure 5 shows the effect of grooves which was made in the samples and in the same time reduces the number of laser pulses.

### 3.4. Surface Morphology Results

**Figure 6:**
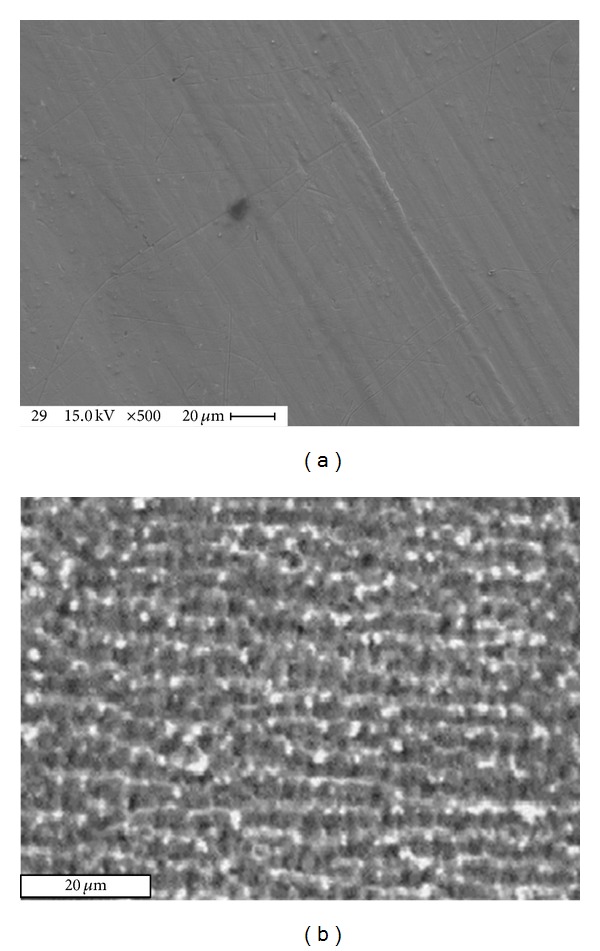
SEM test for aluminum alloy samples: (a) before wear test and (b) after wear test.

**Figure 7:**
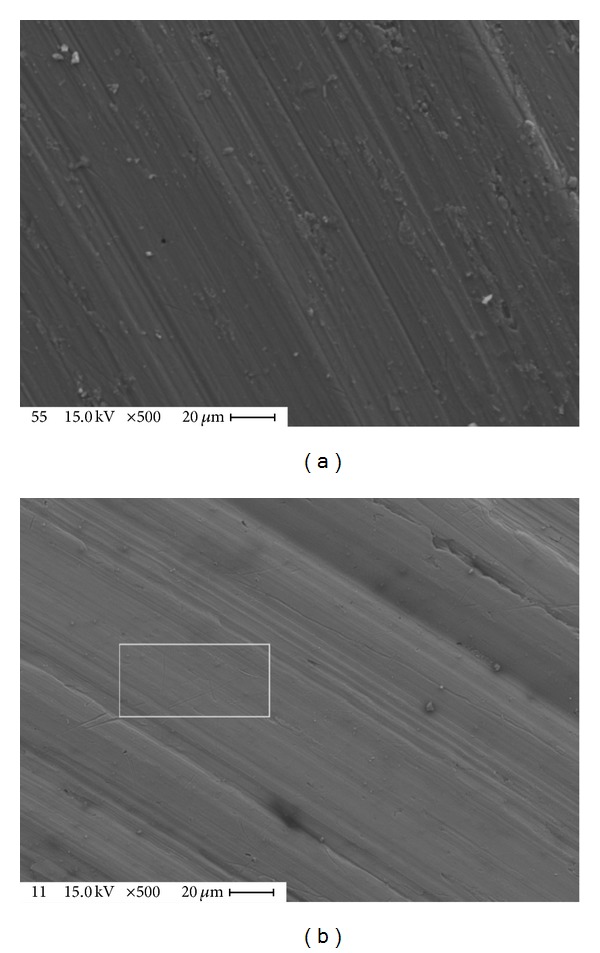
SEM test for aluminum alloy samples: (a) after laser treatment and before wear test and (b) after laser treatment and after wear test.

The surface morphology of aluminum alloy samples was conducted by using SEM technique as shown in Figures 6 and 7. One can notice the differences between the surface samples before and after wear tests. These differences are due to plastic deformation of the surfaces before and after laser treatment. The interpretation of this behavior related to the plasma is an outcome of the gas phase, which absorbs the energy directly from the laser radiation and from the reflection of the material surface. The plasma causes a shock wave by its expansion and this expansion increased by water pressure that covered the sample. For this reason the hardening was increased and grain size decreased.

## 4. Conclusions

Based on the obtained results, we can conclude that laser surface treatment allows decreasing the wear rate by 50% as an approximate ratio when we used 500 pulses and by when we used 250 pulses and grooves. Also, the Vickers microhardness of laser treated aluminum alloy samples is higher than that of the microhardness of the untreated aluminum alloy samples.

## Figures and Tables

**Table 1:** Elemental composition of Al samples.

Comp.	Al	Cl	Ca	Ti	Cr	Mn	Fe	Cu	Te	I	Dy	Er
Conc. (%)	84.5	4.2	0.07	0.07	0.11	1.20	0.75	7.37	1.40	0.07	0.24	0.001
